# Quarantine preparedness – the missing factor in COVID-19 behaviour change? Qualitative insights from Australia

**DOI:** 10.1186/s12889-022-14185-7

**Published:** 2022-09-23

**Authors:** Angela Davis, Stephanie Munari, Joseph Doyle, Brett Sutton, Allen Cheng, Margaret Hellard, Lisa Gibbs

**Affiliations:** 1 Behaviours and Health Risks, Burnet Institute, Melbourne, VIC Australia; 2 Disease Elimination, Burnet Institute, Melbourne, VIC Australia; 3 Department of Infectious Diseases, The Alfred Hospital, Melbourne, VIC Australia; 4 Communicable Disease Prevention and Control, Victorian Department of Health, Melbourne, VIC Australia; 5 School of Public Health and Preventative Medicine, Monash University, Melbourne, VIC Australia; 6 Doherty Institute and School of Population and Global Health, University of Melbourne, Melbourne, VIC Australia; 7 Melbourne School of Population and Global Health, University of Melbourne, Melbourne, VIC Australia; 8 Centre for Disaster Management and Public Safety, University of Melbourne, Melbourne, VIC Australia

**Keywords:** COVID-19, Quarantine, Isolation, COM-B model, Health behaviours, Preparedness, Planning

## Abstract

**Background:**

A key feature of the global public health response to contain and slow the spread of COVID-19 has been community-based quarantine and self-isolation. As part of The Optimise Study, this research sought to understand the factors that influence people’s ability to undertake home-based quarantine and isolation to contain the spread of COVID-19.

**Methods:**

Semi-structured qualitative phone interviews (*n* = 25) were conducted by telephone with people who participated in community-based quarantine in Australia before 31 March 2020. The Capability Opportunity Motivation Behaviour model was used to conduct a thematic analysis.

**Results:**

Participants required clear, accessible and trusted information to guide them in home-based quarantine and isolation. A sense of social responsibility and belief in the efficacy of the restrictions to reduce viral transmission aided their motivation. Access to essential needs, supportive living environments, and emotional support were required to adhere to restrictions, but few were prepared.

**Conclusions:**

Findings demonstrate that in addition to having the capability and motivation to adhere to restrictions, it is vital that people are also encouraged to prepare for the challenge to ensure access to physical, social and emotional support. Findings also illustrate the importance of engaging communities in planning and preparedness for quarantine and self-isolation public health responses.

**Supplementary Information:**

The online version contains supplementary material available at 10.1186/s12889-022-14185-7.

## Background

Novel respiratory virus outbreaks pose a significant threat to public health due to their ability to spread rapidly among populations with little or no prior immunity. A key feature of the global public health response to contain and slow the spread of COVID-19 has been community-based quarantine and self-isolation. According to the World Health Organization; quarantine separates “anyone who is a contact of someone infected with severe acute respiratory syndrome coronavirus 2 (SARS-CoV-2), which causes COVID-19, whether the infected person has symptoms or not” from others; isolation separates those “with COVID-19 symptoms or who have tested positive for the virus” from others [1]. Quarantine and isolation for COVID-19 occurs for a period of approximately 14 days or until public health authorities advise it is safe to leave [2]. Population level cooperation with quarantine and isolation guidelines can reduce the risk of virus transmission and assist contact tracing efforts. Therefore, barriers to initiation and completion can seriously hinder the public health response by accelerating community transmission.

Behavioural science frameworks identify capability, opportunity, and motivation behaviours (COM-B) as key factors which influence people’s response to changes in policy and guidelines such as the introduction of mandatory quarantine or isolation [3]. According to the COM-B model, people require *capability* in the form of knowledge of why a desired behaviour is important and the skills to plan, remember and act on an intention [3]. They need *opportunity* in the form of a supportive or conducive environment in which to practice the behaviours, this includes both their physical surrounds and the wider socio-political and economic context. Finally, behaviours can be *motivated* by existing values or beliefs, a personal belief in the efficacy or importance of a behaviour, its impact on their identity and their ability to overcome existing habits or unconscious processes [4]. Beyond this behaviour system, broader structural factors including the environmental, political, social and cultural contexts can also influence an individual’s experience of quarantine or isolation [5]. Identifying, understanding and responding to barriers and enablers to quarantine and isolation behaviours is critical to developing strategies to enhance participation and reduce adverse health outcomes.

Studies conducted during previous respiratory outbreaks such as SARS in 2003 and H1N1 in 2009, have described barriers and enablers for individuals who are asked to comply with quarantine measures. They identify a lack of communication, confusion, mixed messages and inconsistent information from a number of sources of varying credibility to reducing capability and motivation to quarantine or self-isolate [6–8]. Likewise, knowledge about the outbreak was found to be an important factor as demonstrated by Eastwood et al.where those with a basic level of knowledge of pandemic influenza were more likely to comply with restriction measures [9]. Transparent, timely and evidence-based communication delivered by trust-worthy sources have therefore been identified as key ways to improve capability as well as public trust and allay anxiety [10]. In addition, the level of self-assessed risk of contracting and transmitting the infection can influence an individual’s motivation to engage in subsequent behaviours. Cava et al.found that those who perceived a lesser risk questioned or ignored quarantine protocols compared to those who perceived a greater risk [7]. Proximity to threat emerged as an important factor with some seeing geographical distance and low population density as protective factors, thus reducing motivation to act on public health emergency messages [11].

Motivation to quarantine can be reduced by a fear or loss of employment and income if unable to attend work, concerns about inadequate supplies such as food, requiring medical attention, needing to visit family or to attend religious institutions [12]. Negative psychosocial impacts of the quarantine period resulting in feelings of frustration, boredom, loneliness, fear of stigma and anxiety about contracting or transmitting the infection to others [13] have also been shown to reduce motivation. Support services in the form of flexible psychosocial healthcare and employment options, government assisted financial support and leave entitlements, access to necessities such as medical assistance and groceries and social support groups have all been identified as potential facilitators to quarantine adherence [13, 14]. Additionally, social factors have been found to influence motivation as described by the reciprocity principle or ‘social bargain’ whereby individuals expect their government and society’s assistance in exchange for the loss of liberties assumed during quarantine compliance [15]. Assistance in this sense is not only functional support to create conducive conditions or the opportunity to successfully implement guidelines, but also a source of motivation by demonstrating shared responsibility for community wellbeing.

Quarantine and isolation have been shown to be effective public health strategies for the prevention of COVID-19, especially as there is no proven effective treatment [16]. Particularly in the early stages of the pandemic, when the world was without access to a vaccine, non-pharmaceutical interventions such as quarantine and isolation were, and continue to be, key public health measures in combating the spread of the virus. Despite the development of a vaccine, there will continue to be a need for quarantine and isolation for COVID-19, given the lack of 100% efficacy of vaccines and the potential impact of new viral variants. While literature from previous infectious disease outbreaks has identified factors influencing quarantine and self-isolation behaviours, given the unprecedented scale of the COVID-19 pandemic, there is a need to understand how behavioural factors have influenced quarantine and isolation not only within the COVID-19 context, but also within the wider socio-political and economic contexts. New insights will strengthen our understanding of existing models and enable the advancement of more innovative, self-managed quarantine programs for current and future public health responses.

## Methods

### Setting

This study was conducted between 23–31 March 2020, during the early stage of the pandemic response in Australia. The Australian government closed its international borders on 20^th^ March, 2020 with the exception of returning Australian citizens, residents and immediate family members. All travellers returning to Australia and identified close contacts were required to quarantine at home or in rented accommodation for approximately 14 days or as instructed by the state Department of Health. Diagnosed cases were also required to complete an isolation period of similar duration. A later implemented policy requiring all returned travellers to quarantine in a hotel or designated quarantine was introduced at the end of the study period.

### Theoretical framework

This paper draws on the “COM-B model” of behaviour change [4] to describe barriers and enablers to community-based quarantine and isolation in Australia during the COVID-19 pandemic.

### Study design

This research was conducted as a preliminary phase of The Optimise Study: Optimising Isolation, Quarantine and Distancing for COVID-19 a research project led by Burnet Institute and Doherty Institute that aims to find out how people are experiencing COVID-19 and responding to the measures introduced to stop the spread of the virus. Semi-structured qualitative interviews (*n* = 25) were conducted with people living in Australia to understand their experiences of home-based quarantine or isolation as part of the COVID-19 pandemic response. This study (122/20) and The Optimise Study (333/20) were granted ethics approval by Alfred Hospital Ethics Committee.

### Recruitment

Participants were eligible for the study if they were aged ≥ 18 years, were living or staying in Australia and self-identified as currently or having previously been required to undertake community-based quarantine or isolation before March 31^st^, 2020 for COVID-19. Recruitment was conducted using social media advertising on Facebook and through researcher networks, inviting eligible people to register their interest in the study. Purposive sampling was then used to select final interview participants based on age, gender, languages spoken, location, reason for quarantine and living situation. Invited participants gave informed written consent prior to the interview.

### Sample characteristics

Over 300 people registered interest in the study. From these, 40 were screened using the purposive sampling criteria and invited via email and text message to participate, zero declined and five did not respond to messages, ten people responded after the data collection period had been completed. A total of 25 people participated in the study, each completing in a single interview. At the time of the interview, participants had completed between 2–14 days of quarantine or self-isolation at home while in Australia. Participants were aged between 18–73 years old at the time of the interview. In total 15 participants identified as female, eight as male, two as gender non-binary. Four participants spoke a language other than English at home. Reasons for quarantine or isolation in Australia included returned travel from overseas or interstate (17), diagnosed as a COVID-19 case (3), having close contact with a COVID-19 case (1) and awaiting test results (4). During quarantine or isolation participants lived alone (6), in shared residential accommodation (6) and with a partner or family (13). Two participants reported in the interview that they had completed quarantine in other countries prior to returning to Australia and one participant had been isolated in hospital before returning to complete the rest of their isolation period at home.

### Data collection

A semi-structured interview guide was developed for this study based on a review of existing literature (see Supplementary file). The interview guide was used to prompt and guide a conversation about the experience of quarantine or isolation across identified domains rather than as a questionnaire to be administered. The domains covered quarantine initiation, communication, service provision, daily experiences, understanding and adherence to guidelines and perceived impacts including physical, social, emotional, financial. Interviews were conducted by a female post-doctoral behavioural and social science researcher experienced in qualitative research (AD) who had no prior relationship with interview participants. Immediately prior to the interview, author AD introduced herself, provided information about her professional background and reiterated the reasons for conducting the research. Only the participant and researcher were present for the interviews which lasted approximately one hour and were conducted in English over the phone from the privacy of the author’s home. Interviews were audio recorded, field notes taken during the interview and recordings transcribed verbatim by a professional transcription service. Participants were given the option to review their transcript, though none elected to do so, and all were reimbursed for their time and effort with an AUD$50 voucher.

### Data analysis

A public health framework analysis [17] was conducted using NVivo software. Framework analysis is a thematic analysis process used to identify descriptive findings for rapid translation into policy and practice [17]. First, data were coded by author AD in relation to the three framework nodes—capability, opportunity, and motivation—identified based on the COM-B behaviour change model [3, 4]. Second, data under each node were coded by sub-themes as they arose by authors AD and SM until no new themes were identified. Coding and themes were discussed with the senior author throughout. Major and minor themes presented cover the breadth and depth of data collected with reference to the COM-B framework and quotations are provided to add richer descriptions. Every effort has been made to deidentify the contributions, including removing names and labelling the contributions with identification numbers.

## Results

### Capability

#### Quarantine and isolation capability in a rapidly changing environment

Given the rapidly changing environment in which community members were being asked to quarantine or isolate, participants all reported a need for the provision of a single, trusted information source which explained the responsibilities and rights of people undertaking quarantine or isolation and the latest information on the disease in accessible language. This is illustrated by a participant who said:

*“There’s so much information, what do you trust and what do you believe?”* (ID3)

Participants expressed confusion relating to their rights to exercise outside their property, navigating share-house environments, their risk of transmitting the virus to others and access to strategies to maintain wellbeing throughout the experience, as one participant explained:

*“…I had been given information even if it was a website that said “if you are in self-isolation, this is what it means”. I needed to know explicitly whether I was allowed outside or not. I needed to know what the consequences were if I chose to break the rules, I needed to know how I got access to food and essential medicines if I was on my own. It would have been helpful to have tips on your mental wellbeing when you are stuck at home on how to keep yourself physically active*.” (ID9)

Information overload and mixed messaging forced participants to switch off or turn away from available information and they felt alone in interpreting their ideas about how to quarantine or isolate given their individual circumstances. Circumstances which influenced the type of information required included living environment, language and cultural backgrounds, health needs (including for diagnosed cases, pregnant and post-partum women), family circumstance, access to social supports, mental health issues and employment.

Toward the end of the quarantine period, many participants who were not experiencing symptoms described balancing fluctuating motivation to remain quarantined due to loneliness and anxiety. This was clearly expressed by a participant who explained:

*“I was thinking… in the second week, if I was fine, I might move to a friend’s house and continue self-isolation there…. Like I’m obviously trying to do the right thing, but it’s just based on my judgement. But if there was a way for me to be like “Hey I’m thinking, I wouldn’t mind going for a run at 6am in the morning, is this appropriate to do in self-isolation?” I don’t know where you direct those questions…” (ID1)*

Given potential impacts on mental health, community members may require alternative strategies to relieve negative impacts of the experience as well as information about transmission risk relevant to each day of quarantine or isolation for those seeking to rationalise decisions.

### Motivation

#### Belief in efficacy and ability to impact the control of pandemic

Despite the relatively small number of cases in Australia in the early stages of the pandemic, participants expressed a high level of concern about the seriousness of COVID-19 and a belief in the importance of quarantine or isolation to reduce transmission. When deciding to quarantine, many factored in their perception of risk to themselves and the risk to loved ones, as this participant stated:

*“it was quite a small chance of us having the virus but we know that this is the right thing to do to not expose my in-laws to this”* (ID2)

Risk perception in terms of concern about the seriousness of the disease and belief in the efficacy of quarantine to reduce transmission were both motivating factors. However, many participants were demotivated by a perception that the impact of their personal efforts may be undermined by others’ actions or that they had limited ability to influence pandemic control outcomes, as expressed by participants below who suggested:

*“It’s just really frustrating to see … we live on a main road and the traffic on that road hasn’t decreased since I’ve been home at all, I can still tell people are going out, people are congregating.”* (ID25)

*“you’ve got these professional footballers that had a god damn party on Saturday night…, I’m frustrated the AFL even went ahead because that was pretty spineless move for them to do that and risk the player’s welfare. And just to shit the example for the rest of the country like ‘oh sport can go ahead, and I can go out partying’ or whatever.”* (ID3)

Many participants expressed a sense of social responsibility and self-efficacy during quarantine or isolation but were concerned about the overall efficacy in the context of others. This was described in terms of government, community and individual behaviours and across several different contexts. This illustrates a need to recognise the intersection of individual belief in efficacy and the influence of external factors on motivation when seeking to enhance adherence with isolation and quarantine.

#### Judgement, fear and stigma

Community stigma and judgmental tone in public messaging impacted on both the motivation and opportunity for participants to quarantine as well as on their mental health. This was experienced because of public messaging about individual responsibility to “do the right thing” despite significant structural barriers that community members encountered.

*“… bigger problem is this tone of the information is like, this angry parent… ‘you must do this, if you do this you will be fined’…There’s no messaging that’s like… ‘Hey this is actually something that’s really tough to do, here’s how [to do it]’… it assumes that I’m going to break the rules and like, we’ll get you if you do and…you’re a bad person.” (sic) (ID1)*

Participants from Asian backgrounds experienced stigma and discrimination due to racism. This occurred for adults and children and the experiences made participants less likely to want to disclose their quarantine status to employers, peers or other community members. Some were also fearful about their re-entry into the community after the period of quarantine or isolation. The experience of racism is described by two participants below:

*“she is 10 years old and…the kind of things people at school were saying to her when she came back, like “Chinese people bring the virus to Australia” ...I don’t want to use serious or strong language, but …you know just like that white people are against Asian people who wear the masks….”* (sic) (ID21)

“*my boss never said anything about not being allowed to take sick days, but I guess it’s that sense of I’m not contributing. I also wonder if … because I’m Asian as well and there’s been a lot of racism against Asians since the virus started spreading*.” (ID19)

Stigma due to positive diagnosis was experienced by a range of participants from non-migrant backgrounds due to fear, misinformation or lack of information about re-entry into the community after a positive diagnosis. Some employers and community members had requested doctors’ certificates from people who had completed a 14-day quarantine without symptoms which, at this time during the early stages of the pandemic, was not required.

### Opportunity

#### Active provision of services as a practical and symbolic message of shared responsibility

In general, participants identified a lack of support services as a barrier to following guidelines. While many were able to rely on family and friends to help them, the gap was described as *a “fend for yourself but stay away from others situation” (ID13)* and many were uncomfortable with being a “burden” or questioned the reasonable expectations that they could place on family and friends to provide for them.

*“if everyone’s going into lockdown…like what’s a reasonable expectation for people to support me as well?”* (ID1)

“*Basically we were left to our own devices,…we just had to Google it and kind of look it up ourselves, no one has been in contact with us in terms of being isolated … no government agency or anyone has actually asked us…*.” (ID21)

Participants were highly aware of the changing pandemic response measures and community reaction to them. There was a heightened sense that they were alone to manage often challenging structural issues that impacted on both their opportunity to quarantine or isolate effectively and their motivation due to mental health impacts as expressed by a participant who stated:

*“It’s a huge difference between feeling like you are doing a specific duty to protect people, as opposed to being thrown in a locker and throwing away the key and there’s been no way to…move forward… it’s just the discomfort you have to sit with for a few weeks and there are very few ways to let it out and keep distancing, it’s hard to live. I’ve never felt so disconnected before”* (ID24)

A few participants who, despite understanding the purpose and importance of quarantine and being highly motivated to adhere to these restrictions, reported that they “broke quarantine” because they needed to access food and income support. After unsuccessfully trying to access federal government income support via phone, a few participants left quarantine to attend the support office, while others collected food and medicines from local shops as described by this participant who said:

*“I went to the local supermarket probably in breach of my personal and government rules … I went down there specifically to check what was happening with availability of things…then I went to the chemist shop and got a month’s supply of my medications and checked them for hand sanitizer and medicated soap and they were out of them, so that way I informed myself about what the situation was in that regard.”* (ID7)

While access to food and essential services can provide community members with the opportunity to follow guidelines, the symbolic offer of services may also enhance a sense of shared responsibility for people participating in quarantine and isolation, thereby increasing motivation.

#### Physical environment that promotes mental and physical health and wellbeing

A critical enabler of quarantine and isolation was identified as the physical environment in which people were residing. People with access to their own house with a garden, pets and ability to access outdoor space had significantly less concern about their mental health and their ability to stay at home for the required amount of time.

*“I’ve got a road bike that I’ve put on a wind trainer in my garage… I’ve got a backyard so that’s great, so I’m just throwing a ball around and playing with him [dog]…. I’ve been gardening, …I know that I’ve got people looking out for me and getting good fresh fruit and vegetables, I’ve got a yard to be out in the sunshine and to be able to get outside.”* (ID10)

*“…having a good view. I look out to a park which is very good for my morale rather than just a brick wall, which I think could be quite maddening. Just to have some sort of window view where you can see some greenery or nature because it is quite soothing.”* (ID18)

Conversely, people with less control over their environment and the ability to access communal spaces and outside areas reported higher mental health impacts affecting their motivation and opportunity to continue to quarantine as described by a participant who said:

*“I have a room to myself and basically I have been staying in the room. I eat on a separate table, which is two meters apart. [We are] keeping our distance in the assumption that I have it, which is quite effective for a while, but you know after a while it actually does your head in a bit…. As the days have gone on, I’ve got yet more uncomfortable with the quarantine and you feel you should have had symptoms some time ago, so you become a bit more lax over time (sic)”* (ID24)

The physical environment in which people were undertaking their period of isolation or quarantine had a significant influence on their opportunity to follow guidelines. This indicates that supporting people by providing a more conducive physical environment may improve adherence with these public health requirements.

#### The need for emotional support in a time of uncertainty

The unchartered nature of their situation and ongoing uncertainty about the future had a significant effect on people’s mental health and wellbeing during quarantine and isolation. The feeling of social isolation left many feeling lonely, disconnected and concerned about family members and those less fortunate in the community. Uncertainty around their immediate future created a source of stress and anxiety for many. Many participants were unsure if authorities knew about their situation and expressed that they would have liked to have had someone check in on them, as described by two participants who stated:

*“…No one checked on us whether or not we needed any support. It all 100% relied on our family in Melbourne and friends.”* (ID2)

“*I don’t know the feasibility of it, but just like a check in phone call of someone just sort of just seeing that you’re alright. Or like, ‘what do you need?’ Would’ve been really helpful.” (ID1)*

Many participants identified the desire and need to talk to someone about their difficulties but were unfamiliar with available mental health support services and had little experience accessing them, as one participant stated:

*“I haven’t really seen anything advertising psychological support for people. What if you already suffer from anxiety or depression and you suddenly have to isolate and you know, what if you are having a really bad panic attack or anxiety attack, who’s going to help you then, that’s a worry, so I think more information around supportive services around mental health is important, because that’s going to be what forces people to go outside of isolation”* (ID10)

“…*Your mental state does get impact(ed) by this, if you’re not a strong person it can be quite a lot*…” (ID18)

In the face of few formal support services available, many people sought and utilised their own informal support networks as seen by friends and family members dropping off necessities such as groceries and medications. Several participants also spoke about the emotional support they received by joining online community groups to share their experiences, ask questions and receive and provide offers of assistance, as described by two participants:

*“it’s just like people posting … nice things that they do for other people or what others have done for them … people giving away toilet paper to the elderly and having that connection with them to help people out in times of like need I guess so that’s kind of nice to have that as well. It’s kind of like a positive newsfeed instead of you know, having to worry so much.”* (ID20)

The need for emotional support as a way of increasing the opportunity for people to continue their isolation or quarantine highlights the importance of providing accessible mental health support services and including these in the planning process.

#### Planning and practicing to promote self and community efficacy

In the early stages of the pandemic in Australia, needing to quarantine or isolate was “a shock” to many participants who did not have time to plan strategies to enable this to happen. Quarantine preparedness planning is a strategy which appears to have reduced anxiety about the experience, enhanced motivation and resulted in enabling environments for a few participants. The small number of participants who had developed a plan to guide them were more positive about their ability to overcome any challenges they may face in completing quarantine or isolation. These few participants developed plans which included; how to interact with others in the household, who would support them with groceries and essential items like medication and strategies for maintaining physical and mental health in their context. This was exemplified by one participant who stated:

*“…working out strategies in conversations with my house mate on the phone and letting him talk about anything…I then sent emails about “these are the things we need to do”, because we each had our own bedroom, our own living room, we had the shared spaces, so I kind of worked it out at a point by point, hoping that it would work in practice….So I think for us having a plan …”* (ID5)

Many people expressed a desire for clearer information about their circumstances and an indication of what they can anticipate as a way of assisting with planning and allaying anxiety. In this way, information acts as a form of emotional support by providing reassurance and practical assistance and can reinforce the opportunity to remain in isolation or quarantine. This was described by two participants who stated:

*“…there was no preparation, we were kind of thrown in and then like, “okay, that’s it you are not allowed to go anywhere. You have to figure everything else out for yourself”. (ID20)*

*“I think that it took me by surprise and that was the most difficult part. If I had been prepared for it, I felt that it would have been better…” *(ID19)

These findings demonstrate that a lack of guidance and planning among participants (and government) impacted on their capability, motivation and opportunity to complete quarantine or isolation.

## Discussion

Our findings describe factors which influenced the capability, motivation and opportunity of people to quarantine or isolate at home in the early stages of the COVID-19 pandemic in Australia. As with evidence from previous pandemics, access to clear, trustworthy and timely information about guidelines [18] and practical advice on how to apply these is required for an effective pandemic response. However, information alone is unlikely to enable the effective practice of quarantine or isolation without acknowledging the importance of motivation and opportunity [3, 19]. Despite information about COVID-19 and quarantine and isolation requirements being available, participant awareness of its availability, ease of accessibility and the utility of the information to their situation was not always present. People with high levels of capability and motivation were unable to adhere to guidelines due to structural, social and environmental factors such as access to food and medication and poor mental health. International studies have demonstrated associations between quarantine and isolation and perceived stress and psychological impacts [20, 21].

Importantly, our findings demonstrate the need to revisit recommendations [22] that call for an extension of public health emergency planning beyond technical capability and institutional planning to ensure that communities have the capacity, opportunity and motivation to respond. It was perceived that at a community level, a lack of planning and ongoing uncertainty could result in individuals who had the desire and capacity being unable to follow them. Opportunities to plan for and practice strategies that people may need during their experience and linking them in with existing services and their own community networks may improve adherence and health outcomes. Consistent with natural disaster emergency planning and response, community-wide planning is a mechanism to engage community members, improve their capability to act and create a sense of self and community efficacy by establishing an environment that supports planned behaviours [23]. In the case of rapid COVID-19 policy creation and responses, prior planning, preparedness and community engagement may have led to an already established sense of self-efficacy and hence improved resilience. This may not be useful or practical for all community members, however it will be for some. This could result in increased capacity for government to focus on providing intensive and targeted support to those who are less able to plan for and respond to the need to self-quarantine or isolate. The process may address some structural barriers experienced by people attempting quarantine and isolation and create a better sense of shared responsibility to motivate ongoing community wide participation in pandemic response measures [15, 24].

Our findings suggest that supportive public messaging, which demonstrates the efficacy of quarantine and isolation and normalising the behaviour, would enhance a sense that community members are seen, supported and valued for their participation. While risk perception and belief in the efficacy of quarantine and isolation can be important motivators to follow guidelines [7], this motivation may be reduced if community members feel as though their actions are in vain because of ongoing transmission or a perceived lack of shared responsibility. This highlights a sensitive balance required in the communication of risk and epidemiological trends during the COVID-19 pandemic. Clearly there is a need for governments to communicate the ongoing risk of transmission and the impacts of non-adherence to guidelines. However, other research suggests that focusing on calling out undesired behaviour is a less effective behavioural strategy than promoting, normalising and rewarding the desired behaviour to influence social norms [19]. Positive and supportive messaging can also enhance the mental health of people during quarantine which has also been shown to impact motivation to follow guidelines [13]. In addition to improved information on self-isolation, the provision of social support and clinical intervention to improve emotional wellbeing is likely to improve rates of adherence [25].

Importantly our findings indicate that racism and discrimination impact the experience of quarantine and isolation. This is consistent with previous research highlighting the negative impacts of quarantine including reinforcing stigma against social minority groups [26]. The fear and threat that results from discrimination can not only affect a person’s identity but can also affect attitudes to others, undermining empathy with those who are undertaking physical distancing measures [27]. These impacts on identity and existing values and beliefs can play a significant role in the motivation to adhere to isolation and quarantine requirements. Further research and engagement with diverse community members is needed to identify and define key barriers and enablers to enhance the ability to follow guidelines and reduce negative impacts of the measures [28]. This includes continuing to engage and support community members in testing and quarantine procedures, even if fully vaccinated against COVID-19.

Our findings clearly demonstrate that factors related to capability, opportunity, and motivation [3] intersect to influence quarantine and isolation behaviour. They support an expanded focus beyond information and public messaging that only address capability and motivation, to enhance community planning and preparedness. This helps to ensure that policy and planning create enabling environments, addressing limitations of physical living circumstances; supporting provision of essential goods and services; and addressing mental health, stigma and discrimination that may result in population level challenges to effective quarantine and isolation [18]. Understandably, it is difficult to influence the current physical environment of all community members who may need to quarantine and isolate in high density areas common to cities. However, this should influence the development and targeting of specific messaging and support services to people based on their physical environment and has potential long-term implications for urban planning. An example of this is the subsequent implementation of an emergency accommodation program in Victoria for community members who are unable to quarantine or isolate safely at home [29]. Working with community members to develop and implement household level quarantine and isolation plans that connect them with services prior to their need arising, may enable people to access necessities during the experience, alert community organisations to people’s needs and promote an environment of shared responsibility.

### Strengths and limitations

The in-depth data captured in these interviews from people in diverse circumstances provide valuable insights into experiences of quarantine and isolation. Alignment of the findings with the Capability Opportunity Motivation Behaviour model also contributes to consideration of the practical applications. However, this should be accompanied by caution in recognition of the study and sample limitations which include: recruitment conducted online and through researcher networks limiting the sample to technology confident groups, though in doing so, actually enhancing our insights into a group who were more likely to be impacted by the available information and communication; recruitment and data collection conducted only in English limiting the number of culturally and linguistically diverse (CALD) communities; single phone interviews for each participant, at various times during and after their quarantine or isolation experience; conducted during the early stages of Australia’s pandemic response when little was known or could be planned for in relation to the severity or scale of the pandemic. As such, the relevance of the application of our findings to groups not included should be noted with caution.

## Conclusions

Findings from this study conducted during the early stage of Australia’s pandemic response provides unique insights into ways to support community-based quarantine and isolation. Household and local community planning for quarantine and isolation may enhance community-wide capability, motivation and opportunity and reduce negative impacts of the experience. Findings presented in this paper can be used to enhance support for quarantine and isolation in Australia and globally. Comprehensive community engagement strategies such as co-design will enhance the design of strategies that support communities to participate in infectious disease control measures.

## Research implications

Using a behavioural science framework, this study provides an in-depth understanding of the behavioural and broader contextual influences on home quarantine and isolation experiences during the early stages of the COVID-19 pandemic. Findings can be used to inform current and future pandemic preparedness and planning by public health practioners and policy makers to mitigate the adverse health impacts of future pandemics.

## Supplementary Information

**Additional file 1.**

## Data Availability

Authors can confirm that all relevant data are included in the article. The datasets generated and/or analysed during the current study are not publicly available due to them containing information that could compromise research participant privacy/consent. However, data are available on reasonable request and with permission from the corresponding author (SM) and Burnet Institute.

## Abbreviations

CALD: Culturally and linguistically diverse
COM-B: Capability opportunity motivation behaviour
COVID-19: Coronavirus disease 2019
SARS-CoV-2: Severe acute respiratory syndrome coronavirus 2
