# Supplementary material for: Quarantine preparedness – the missing factor in COVID-19 behaviour change? Qualitative insights from Australia
Source: BMC Public Health. 2022 Sep 23;22:1806. doi: 10.1186/s12889-022-14185-7 (PMC9502922; doi:10.1186/s12889-022-14185-7)
Supplement: Supplementary file 1 — Additional file 1. [file 12889_2022_14185_MOESM1_ESM.docx]

# Phase 1. Interview Guide

## Section One: Quarantine initiation and compliance

1. To start our discussion would you mind telling me about your situation starting with why did you first quarantine and when was that? (how long you’ve been in quarantine), your living arrangements, and whether anyone else lives with you? Do you have any dependents? E.g. children, elderly, people you provide care for, pets?
2. Talk me through a regular day since you have been in quarantine from when you wake up to when you go to bed?

## Section Two: Assessment of the individual’s experience of self-quarantine and identification of positives, issues and concerns (sections to cover if not mentioned spontaneously)

### General areas to cover

1. How has your experience been so far?
   1. What do/did you like about your quarantine experience?
   2. What do/did you not like about your quarantine experience?
2. What issues have most worried you about having to be quarantined?

Probe for:

1. Your health
2. Transmitting to others
3. Unable to access products and services
4. Unable to earn money
5. Unable to see friends/family
6. Fear or stigma or discrimination (being treated differently)
7. Did you go outside during your quarantine period? If so, and if you’re comfortable telling me, why did you leave the space you were quarantined to? Did you need to leave your location for any reason? If so, for why?

### Income- domain

1. What do you do for a living? I.e. working, studying or unemployed?
2. From where do you normally get your income?
3. Have you been able to work/study during your quarantine time? If not, have you been able to find an alternative such as working from home? Has your income been affected due to the quarantine period? If so, in what way and have you found alternative sources of income during the quarantine period?
4. If you’ve been unable to carry out your normal work/study, have you been concerned about this?
5. Have you received any support from your place of work/study?

### Necessities-domain

1. Have you been accessing food for meals during your time? If yes how, if not, how did you manage this?

Probe for:

- 1. Use of meal delivery services or online shopping
  2. Reliance on community support
  3. Adequacy

1. What about other household items?

Probe for personal hygiene supplies such as toilet paper, soap, toothpaste, feminine hygiene products? If not, how did you manage this?

1. Did you have access to the internet during your time? Did you have enough data?
2. Did you use any transport during your quarantine period? Which form of transport?

### Health- domain

1. Do you feel your physical health was impacted, either positively or negatively, by the quarantine period? If so, how?
2. Do you feel your mental health was impacted, either positively or negatively, by the quarantine period? If so, how?

Probe for:

- 1. Positive impacts: more relaxed, calm, happy
  2. Negative impacts: boredom, frustration, anxiety, fear, sadness, loneliness

1. Do you have access to all/any of the medications that you need?
2. Have you been exercising? If so, what kind of exercise and how have you been doing it?
3. What would you do (who would you call or where would you go) if you were worried about your health?
4. What kind of things have you been doing to help you manage with any of the identified problems above? i.e. coping strategies

### Social connectedness-domain

1. Who have you been in contact with during your quarantine experience? Or other quarantined people? If so, how?
2. Have you been able to engage with your usual social/community groups? E.g. church, volunteering. How has this affected you?
3. Do you feel connected with your local community and the general public?
4. Are you aware of any social groups with other quarantined people that you can connect with?
5. How could we improve your connectedness with those around you?

### Other issues and feedback

1. Have you come across any other issues or concerns before, during or after your quarantine period that we haven’t covered?
2. What more do you feel the government could be doing or providing you with to help you and your family before, during and after this quarantine period?
3. Do you have any feedback on any aspect of the quarantine process?
4. Do you have any questions for me before we finish today?

## Section Three: Understanding of Covid19 and quarantine initiation and compliance

1. Even though we have talked about it a bit, I would like to ask more about what you know about the about Covid19 (previously termed novel coronavirus 2019) outbreak- can you tell me a bit about what you think the virus is?

Probe for knowledge of:

- 1. Seriousness of outbreak
  2. Transmission
  3. Signs and symptoms
  4. Management principles

1. What’s your understanding of your requirements for quarantine?

Probe for:

- 1. Purpose of quarantine
  2. Length of quarantine period
  3. Contact with other people

1. What information have you been given about quarantine? And has this been made clear to you?
2. Where have you been looking for information on this topic? i.e. government websites, community forums
3. What support services or contacts are you aware of if you have any questions/issues?

Thank you for your time and for providing us with this valued information. Here is a list of support services which we give to all participants in case this interview raised any questions or concerns that you would to speak about.
